# Differentiating surface titanium chemical states of anatase TiO_2_ functionalized with various groups†
†Electronic supplementary information (ESI) available. See DOI: 10.1039/c7sc04828a


**DOI:** 10.1039/c7sc04828a

**Published:** 2018-01-29

**Authors:** Yung-Kang Peng, Hung-Lung Chou, Shik Chi Edman Tsang

**Affiliations:** a Department of Chemistry , University of Oxford , OX1 3QR , UK . Email: edman.tsang@chem.ox.ac.uk; b Graduate Institute of Applied Science and Technology , National Taiwan University of Science and Technology , Taipei 10617 , Taiwan . Email: HLCHOU@mail.ntust.edu.tw

## Abstract

As the chemical state of titanium on the surface of TiO_2_ can be tuned by varying its host facet and surface adsorbate, improved performance has been achieved in fields such as heterogeneous (photo)catalysis, lithium batteries, dye-sensitized solar cells, *etc.* However, at present, no acceptable surface technique can provide information about the chemical state and distribution of surface cations among facets, making it difficult to unambiguously correlate facet-dependent properties. Even though X-ray photoelectron spectroscopy (XPS) is regarded as a sensitive surface technique, it collects data from the top few layers of the sample, instead of a specific facet, and hence fails to distinguish small changes in the chemical state of Ti imposed by adsorbates on a facet. Herein, based on experimental (chemical probe-assisted NMR) and theoretical (DFT) studies, the true surface Ti chemical states associated with surface modification using –O–, –F, –OH and –SO_4_ functional groups on the (001) and (101) facets of anatase TiO_2_ are clearly distinguished. It is also demonstrated, for the first time, that the local electronic effects on surface Ti imposed by adsorbates vary depending on the facet, due to different intrinsic electronic structures.

## Abstract 2

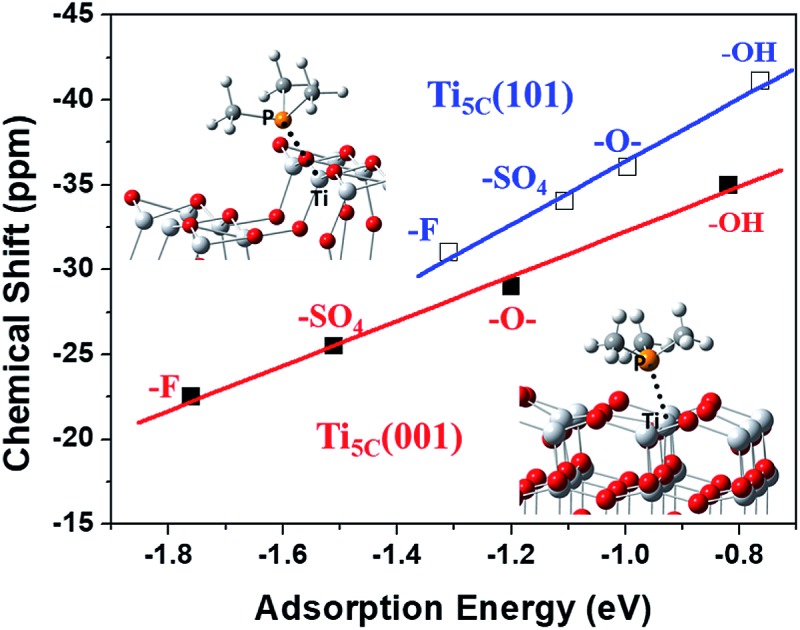
The local electronic effects on surface Ti, caused by adsorbates on TiO_2_ facets, are probed experimentally (using probe-assisted NMR spectroscopy) and theoretically (using DFT).

## Introduction

The facet stability and reactivity of inorganic single crystals have long been thought to be dominated by their surface chemistry, which has a critical effect on the equilibrium morphology in the preparation of faceted nanoparticles (NPs) with more superior properties.^1^,^2^ Structural directing species (SDS) (*e.g.* surfactants) employed during the control of morphology, which enable the synthesis of NPs in metastable and high-energy forms, are usually removed to avoid their interference with the interesting properties related to facet activity.^3^ In particular, in heterogeneous catalysis involving the breakage and formation of bonds at a particular catalyst surface, the nature of the surface is closely associated with the coordination environment of the surface features (*e.g.* oxygen vacancies, hydroxyl groups, metal cations, SDS residues, *etc.*). Techniques such as photoluminescence (PL) spectroscopy, Raman spectroscopy, electron paramagnetic resonance (EPR) spectroscopy and X-ray photoelectron spectroscopy (XPS) are currently employed for the characterization of surface features, from the top few layers to the bulk, however, no information about specific facets is available.^4^–^6^ They actually provide very limited information about the topmost chemical states and their distribution among facets, causing ambiguities in correlating facet-dependent properties and thus leading to different interpretations amongst researchers.^5^,^6^

Here, anatase TiO_2_ is taken as an example, which is one of the most studied metal oxides due to its versatile applications. The (001) facet of anatase TiO_2_ has long been expected to be more catalytically active than the (101) facet, while its higher surface energy (0.90 J m^–2^) (*cf.* 0.44 J m^–2^ for the (101) facet) makes it difficult to synthesise at higher coverage.^7^ The breakthrough in the synthesis of anatase TiO_2_ with the preferential exposed (001) facet was achieved by Yang *et al.*^8^ in 2008, using fluoride as a SDS to form Ti–F on the (001) facet, which reverses the relative thermodynamic stability of the two facets. Using the SDS strategy, the introduction of a surface group in this case, fluoride was first used for the synthesis of the higher energy facet, followed by its removal by either calcination in air at 600 °C (ref. 9–17), or ion exchange with aqueous NaOH,^12^,^18^–^23^ to obtain a so-called “clean surface” in the meta-stable form prior to catalytic applications or further modifications. However, by adopting different F removal methods, diverse results and disagreements have been obtained among researchers.^9^–^23^ For example, calcination treatment in wet air enables the replacement of surface Ti–F by Ti–OH, as shown by XPS, but this treatment was sometimes reported to be accompanied by particle aggregation along the [001] direction with a reduction of the (001) facet, and may also induce the reconstruction of rest (001) facet.^24^,^25^ In contrast, no induced aggregation and reconstruction were reported after washing with NaOH, while the F removal was suspiciously incomplete, so presumably the F residues offered the facet stabilisation.^6^,^22^,^23^ So far, no clear rationalization or guidance for the selection of appropriate post-treatment methods has been achieved. This raises concerns, such as: “what happens to those high energy facets before/after the removal of SDS?” and “do they still remain the same?” The answer to these questions is crucial for filling the gap between the model catalysts used in surface science and the real catalysts found in practical applications.

Moreover, it has also been reported that an adsorbed molecule, if retained on the TiO_2_ surface, may modulate the chemical state of the surface Ti, the physiochemical properties of which may therefore deviate from those of the clean surface shown in calculations. For example, the attachment of PO_4_^3–^ (ref. 26)/SO_4_^2–^ (ref. 27) onto the TiO_2_ surface is reported to provide extra Brønsted acid (BA) sites and at the same time increase the Lewis acidity of the exposed Ti atoms. Also, the chelation of COOH-containing dyes^28^ and electrolyte additives such as 4-*t*-butylpyridine (TBP)^29^ has been found to remarkably improve the solar cell performance due to a shift of the Ti d-band edge toward negative potentials due to adsorption onto the TiO_2_ surface. Such surface modifications could be very different from facet to facet, but no study has yet been reported in the literature. The main problem delaying the important rationalization of facet-dependent properties so far is the lack of a reliable characterization tool that is sensitive enough to reflect the local change in the chemical state of Ti on different facets that is promoted by adsorbed molecules.

It has been shown that nuclear magnetic resonance (NMR) spectroscopy is a powerful and sensitive technique that can be used to differentiate a small change in the chemical state of surface features among facets, through the adsorption of NMR-active chemical probe molecules.^4^–^6^ Here, we demonstrate that by using this technique, in combination with theoretical (DFT) studies, the small changes in the chemical states of surface Ti are differentiable and dependent on the electronic effects exerted by surface groups (–O–, –F, –OH and –SO_4_). It is also demonstrated for the first time that such surface modifications by residue surface groups occur to different extents, which depend on the nature of facet exposed.

## Results and discussion

### Preparation and characterization of TiO_2_ samples

To study the effect of Ti–F on the surrounding Ti atoms on various facets, TiO_2_ samples with different amounts of the (001) facet exposed were prepared according to the literature by adding HF during hydrothermal synthesis.^8^,^9^ Samples prepared with increasing volumes (0 mL, 2 mL and 6 mL) of 50 wt% HF have different morphologies, and are denoted 0HF (Fig. S1a†), 2HF (Fig. S1b†) and 6HF (Fig. S1c†). All samples showed lattice fringes with *d*-spacings of around 0.35 and 0.47 nm, corresponding to the [002] and [101] crystallographic plane directions of anatase TiO_2_, respectively (also see the XRD results in Fig. S2†). The percentages of the (001) facet exposed in the 0HF, 2HF and 6HF samples were estimated, according to the Wulff construction model (Fig. S3†), as 10.2%, 21.1% and 75.4%, respectively, from their TEM images (Table S1†) despite some degree of surface irregularity and corrugation. It is noted that the 0HF sample was characterized with ∼90% (101) facet, which matches well with the dominant thermodynamic stability of the (101) facet in anatase TiO_2_, as predicted by the Wulff construction (∼94%).^30^

### Comparison between the Raman, XPS and NMR results

**Fig. 1:**
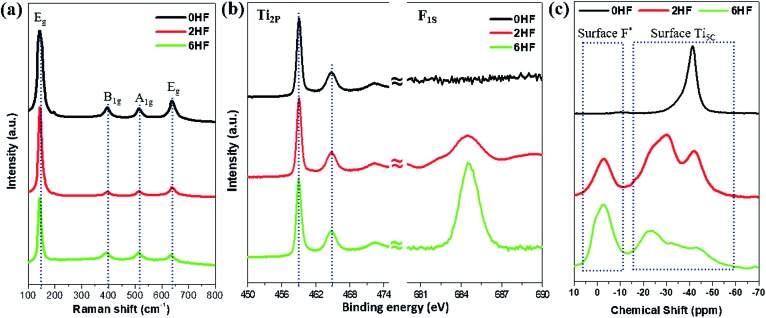
(a) Raman and (b) XPS Ti_2P_/F_1S_ spectra of 0HF, 2HF and 6HF samples. (c) ^31^P NMR spectra of trimethylphosphine (TMP)-adsorbed 0HF, 2HF and 6HF samples. *The acidic proton induced by surface F.

Raman spectroscopy^11^,^15^,^31^,^32^ and XPS^8^–^16^,^22^,^23^ have been widely employed in the literature as surface tools to monitor the existence of fluorine attached to the surface. For Raman characterization, it has been shown that the fluorine attached to the surface changes both the “symmetry of Ti–O–Ti” and the “coordination of surface Ti atoms”, resulting in a “shift of the low-frequency E_g_” and a “strengthening of B_1g_ (*cf.* A_1g_)”. However, from our experimental results, only a marginal shift of the low-frequency E_1g_ is observed for the samples with HF added (*i.e.* 2HF and 6HF) (Fig. 1a). XPS is a generally accepted technique to monitor element(s) on the surface of a material. As shown in Fig. 1b, the F_1S_ signal increases with an increased amount of HF added during preparation (6HF > 2HF > 0HF) as previously reported,^6^,^11^,^22^,^23^ but no chemical shift of the Ti_2P_ signal can be observed in the presence of fluorine attached to the surface.^6^,^22^

Alternatively, chemical probe-assisted NMR has recently been employed for the characterization of the electronic interactions in solid structures, as it can reflect the various microenvironments of solid surfaces using chemical shift values that change due to adsorption of the probe molecules.^33^ Among NMR-active probes (*e.g.* carbon monoxide for ^13^C, pyridine for ^15^N, trimethylphosphine (TMP) for ^31^P, *etc.*), TMP containing ^31^P nuclei with 100% natural abundance and a chemical shift range over 430 ppm has been shown to be a sensitive and reliable chemical probe species that can provide both qualitative (strength) and quantitative (concentration) information about the discrete acid sites in various acid catalysts, such as microporous zeolites, mesoporous molecular sieves and supported/sulfated metal oxide catalysts, through the adsorption of this basic molecule.^33^ As shown in Fig. 1c, using TMP as a chemical probe to interrogate surfaces using ^31^P NMR not only monitors the surface F content (*via* the acidic proton) but also provides peak shifts at high resolution, which occur due to the electronic change of Ti imposed by this SDS (discussed below). Regardless of the physicochemical meaning of each peak, this probe-assisted NMR indeed provides extraordinary sensitivity to the chemical states of surface features, compared to the traditional surface tools mentioned above.

As stated, calcination treatment^9^–^17^ and washing with NaOH^12^,^18^–^23^ are the two commonly used methods reported in the literature to remove surface F from F-treated samples after the control of TiO_2_ particle morphology. However, as evidenced by TEM images (Fig. S4†), calcination treatment causes severe particle aggregation for all of the three as-prepared 0HF, 2HF and 6HF samples, while washing with NaOH doesn’t lead to any observable aggregation or change in morphology. This result can be further supported by the observation of sharpened XRD signals (*i.e.* increase of crystallinity, Fig. S5†) and increase in the particle size (Table S2†) of the calcined samples. Accordingly, to study the –OH and –SO_4_ modified TiO_2_ surface without interference from the crystallinity and size, washing with NaOH and subsequent sulfation for a prolonged time periods were carried out on the as-prepared 0HF, 2HF and 6HF samples (see the Experimental section for details). Compared to the results from probe-assisted NMR (Fig. S6†), both Raman (Fig. S7†) and XPS Ti_2P_ (Fig. S8†) measurements failed again to distinguish “the change of the Ti vibration frequency” and “the chemical shifts of Ti_2P_” that were induced by the –O–, –F, –OH and –SO_4_ groups on both the (001) and (101) facets at high coverage after extensive surface treatment. Even though XPS has been regarded as a surface sensitive technique to probe atomic chemical states, the long electron escaping depth (few atoms deep) for samples studied by XPS means that it is not a true surface technique, and the detection of core-electrons also makes the binding energy of Ti less sensitive to electronic effects from neighbouring adsorbates.

### NMR results for TiO_2_ samples after various treatments

**Fig. 2:**
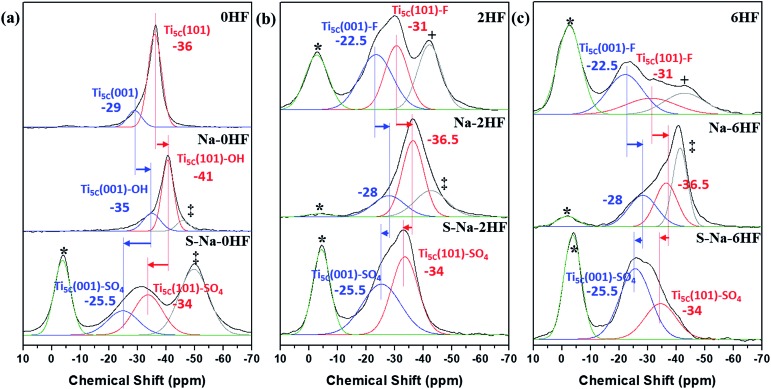
^31^P MAS NMR spectra of TMP-adsorbed (a) 0HF, (b) 2HF and (c) 6HF samples (first row), after washing with NaOH (second row, *i.e.* Na–0HF/2HF/6HF), and after subsequent sulfate modification (third row, *i.e.* S–Na–0HF/2HF/6HF). See ref. 6 for the detailed assignment of the *Brønsted acid site, +surface oxygen vacancy and ‡Ti_5C_ (101)–OH.

**Scheme 1:**
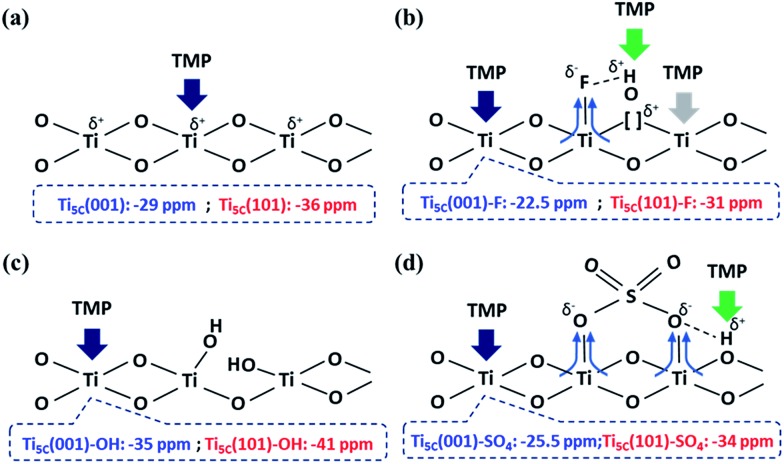
An illustration of the interactions between TMP and the surface features on TiO_2_ facets, promoted by (a) –O–, (b) –F, (c) –OH and (d) SO_4_^2–^ groups.

In general, the chemical shift of ^31^P (δ^31^P) in the range of –2 to –5 ppm is attributed to the formation of a TMPH^+^ ionic complex (*i.e.* a Brønsted acid site), while the δ^31^P of adsorbed TMP spans over a wide range (–20 to –58 ppm) when interacting with surface exposed metal ions (*i.e.* Lewis acid sites) with various Lewis acidities (Fig. S9†).^33^ In this wide range, the stronger TMP–Ti bond formation pushes δ^31^P downfield and thus differentiates various Ti chemical states by their corresponding chemical shifts. Fig. 2 shows the corresponding ^31^P NMR spectra of the TMP-adsorbed 0HF, 2HF and 6HF samples (first row), after washing with NaOH (second row), and after subsequent sulfation (third row). The 0HF sample shown in the first row of Fig. 2a reveals only a TMP-LA signal with the main peak at –36 ppm and a small shoulder at –29 ppm (a typical standard measurement deviation of ±1 ppm in the chemical shift position was collected). Similar results for chemical shift values have been reported by Deng and co-workers over their titanium oxide prepared without HF.^27^ The major peak at –36 ppm and the shoulder at –29 ppm, with an integrated area ratio of 84.8% and 15.2%, can be attributed to the interaction between TMP and the surface five-coordinate Ti atom on the (101) facet (Ti_5C_ (101)) and the (001) facet (Ti_5C_ (001)), respectively (Scheme 1a). The high proportion of the (101) facet in the 0HF sample also matches very well with the prediction from the Wulff construction, taking estimation error into account (89.8%, Table S1†).^30^ For samples prepared with HF (*i.e.* 2HF and 6HF), a 5 and 7 ppm downfield shift from –36 ppm for Ti_5C_ (101) and –29 ppm for Ti_5C_ (001) in the 0HF sample, to –31 ppm (Ti_5C_ (101)–F) and –22.5 ppm (Ti_5C_ (001)–F), respectively, can be observed (the first row of Fig. 2b and c) due to the strong electron withdrawing effect that fluorine exerted on the surrounding Ti atoms on these two facets (thin blue arrow, Scheme 1b). Also, the first appearance of BA sites (∼2.5 ppm) on both the 2HF and 6HF samples (*cf.* 0HF, the first row of Fig. 2) can be rationalized by the surface hydrogen bonding stabilization of the protons by the fluorine (green arrow, Scheme 1b). As supported by XPS F_1S_ (Fig. 1b), the stronger BA signal of the 6HF sample, compared to that of the 2HF sample, indicates that the concentration of acidic protons is proportional to the amount surface-attached fluoride.

The effect of washing the TiO_2_ surface with NaOH was also studied for the as-prepared 0HF, 2HF and 6HF samples (second row of Fig. 2). For the 0HF sample, washing with NaOH clearly reduces the Lewis acidity of Ti_5C_ (001) and Ti_5C_ (101) (0HF, Fig. 2a), as δ^31^P shifts from –29 ppm to –35 ppm (Ti_5C_ (001)–OH) and from –36 ppm to –41 ppm (Ti_5C_ (101)–OH) (Na–0HF, Fig. 2b) due to surface hydrolysis, which causes the breakage of a Ti–O–Ti bond and the formation of two Ti–OH bonds (Scheme 1c). However, different LA distributions are obtained after the same treatment of both 2HF and 6HF (Na–2HF and Na–6HF in Fig. 2b and c). The tiny BA signal between –2 and –5 ppm clearly indicates there are F residues left on the surface of both the Na–2HF and Na–6HF samples.^6^ These residual Ti–F groups, together with those converted to Ti–OH, can result in a very different electron density of Ti_5C_ atoms compared to in 0HF (no surface F, the first row of Fig. 2a) and Na–0HF (surface Ti–OH, the second row of Fig. 2a). This mixed surface functionalization of both F and OH shifts the δ^31^P of TMP-adsorbed Ti_5C_ (001)–F/Ti_5C_ (101)–F from –22.5 ppm/–31 ppm (first row of Fig. 2b and c) to –28 ppm/–36.5 ppm (second row of Fig. 2b and c). Note that no characteristic surface reconstruction of the ^31^P signal at –50 ppm (ref. 6) can be observed for all the NaOH washed samples (second row of Fig. 2); this clearly supports the fact that NaOH treatment, without the application of heat, does not induce extensive reconstruction, compared to calcination treatment. As the sulfated TiO_2_ sample has been reported to have higher Lewis acidity,^27^ samples that underwent sulfation for a prolonged time after the NaOH wash were also evaluated (*i.e.* S–Na–0HF/2HF/6HF in the third row of Fig. 2). Indeed, two new stronger LA peaks, namely –25.5 ppm (Ti_5C_ (001)–SO_4_) and –34 ppm (Ti_5C_ (101)–SO_4_), were introduced for all three samples, due to TMP adsorption on the sulfated surfaces. These two new LA sites arise from the formation of TMP–Ti bonds next to the newly formed bidentate interaction of SO_4_ and the TiO_2_ surface, during the sulfation (thin blue arrow, Scheme 1d). Also, a BA site (∼3 ppm) (third row of Fig. 2) was introduced for the first time to the S–Na–0HF sample, and re-introduced into the S–Na–2HF and S–Na–6HF samples, as it was previously removed by the NaOH wash (green arrow, Scheme 1d).

As a result, the chemical state of Ti induced by –O–, –F, –OH and –SO_4_ groups on both the (001) and (101) facets can be readily distinguished using this technique, with the downward shifts ranked in the order of –F > –SO_4_ > –O– > –OH (*cf.* XPS in Fig. 1b and S8†) under the same conditions. It was reported by Boles *et al.* that a surface-bound molecule generates an electric dipole and this intrinsic dipole depends on its binding mode and chemical structure.^34^ For Lewis-basic molecules, the interfacial dipole points from the ligand towards the Lewis-acidic metal (L^δ–^ → M^δ+^) and therefore modifies the electronic structure of the metal ion. Although there might be errors in the measurements (the standard deviation in NMR measurements) and the presence of defects and impurities, our experimental results are clearly supported by this electronic effect, which states that the largest ligand-induced downward shift of the electronic energy levels is caused by halide ions (*i.e.* fluoride in our case).^34^

It is expected that the degree of surface modification and resulting electronic effects induced by the surface modification should be dependent on the HF concentration and F coverage (see the ESI† for the coverage calculations for surface F and SO_4_). As seen from Table S4,† although the coverage and the BA signal are progressively increased at higher HF concentrations (although the increase is not linear with HF concentration from 2HF to 6HF) the total surface acidity, particularly the LA sites, was found to decrease upon the use of higher HF concentrations. This suggests that the complex F coverage effects not only offer electronic withdrawing properties for the neighbouring Ti_5C_ sites, but also cover or block the same sites, attenuating the total LA acidity value. Fig. S10a† clearly shows the significant change in the chemical shift when F coverage increases from 0% to 20.8% by adding 2 portions of HF during the preparation. The introduction of BA sites and widening of the LA acidity range are clearly visible. However, if the surface coverage is further doubled to 43.0%, by adding 6 portions of HF during the preparation (black line in Fig. S10a†), there is no significant further change to the LA range, but the relative intensity of the peaks decreases (due to a decrease in the population, due to the F blockage of the Ti_5C_ sites), as stated. A similar result is also noted in the case of SO_4_. A wider chemical shift range from ∼15 ppm (green line in Fig. S10b†) to ∼25 ppm (red/black line in Fig. S10b†) was obtained after SO_4_ modification, and no significant further change of the shift range, but a decrease in the relative intensity of the peaks with increased SO_4_ coverage, was observed. It is not yet clear whether the insensitivity of the chemical shift value beyond 2HF is related to the surface symmetry of the position of the adsorbates or other factors. Thus, a study of the effect of the electron affinity of adsorbates (*i.e.* F > SO_4_ > OH) at low coverage was first performed in this work. The effect of adsorbate coverage appears to be complex and will be explored further at a later time. Besides, it is noted that adding HF during preparation not only increases the surface F coverage but also changes the shape of the particles (*i.e.* it changes the population of Ti_5C_ on the (001) and (101) facets), thus making the elucidation of the coverage-induced electronic effects rather difficult.

### DFT calculation

**Fig. 3:**
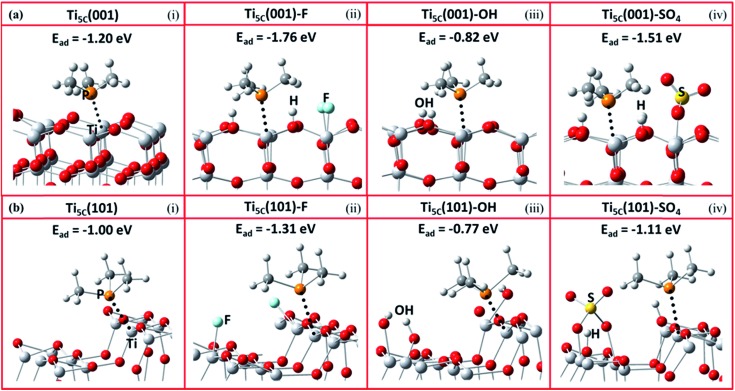
Schematic illustrations of the molecular interactions and the DFT calculated adsorption energy (*E*_ad_) between TMP and Ti_5C_ on TiO_2_ (a) (001) and (b) (101) facets, promoted by (i) –O–, (ii) –F, (iii) –OH and (iv) –SO_4_ groups (Ti: light grey; O: red; P: orange; C: grey; H: white; S: yellow; F: turquoise). Also see Fig. S11 and S12† for the top view of these molecular interactions on the (001) and (101) facets.

It is expected that the stronger Lewis acid–base interactions between the adsorbate and surface Ti give stronger surface adsorption. To confirm the effects of various adsorbates on the chemical states of the surface Ti among the facets, we then carried out a DFT investigation on –O–, F–, –OH and –SO_4_ attached to the (001) and (101) facets, and calculated the corresponding adsorption energies (*E*_ad_) of TMP using the projector-augmented waves generalized gradient approximation (PAW-GGA) and linear response method. Both methods gave the same *E*_ad_ on both the (001) and (101) facets (Tables S5 and S6†). Fig. 3 shows the side view of these molecular interactions on the (001) and (101) facets (also see Fig. S11 and S12† for the top view). For the (001) facet, the *E*_ad_ between TMP and Ti_5C_ on the clean (001) facet is greatly increased from –1.2 eV (Fig. 3a(i)) to –1.76 eV (Fig. 3a(ii)) when the neighbouring Ti_5C_ is fluorinated. This can be supported by our experimental result of a 7 ppm downfield shift, from –29 ppm for Ti_5C_ (001) in the 0HF sample to –22.5 ppm for Ti_5C_ (001)–F in both the 2HF and 6HF samples (the first row of Fig. 2). A similar increase in the *E*_ad_ (from –1.2 to –1.51 eV, Fig. 3a(iv)) and a downfield shift in the experimental chemical state (from –29 to –25.5 ppm, the third row of Fig. 2) can be observed also for the sulfated (001) facet (Ti_5C_ (001)–SO_4_) (*cf.* clean (001) facet). Both cases support the reported downward shift of the electronic energy levels induced by the Lewis-basicity of the adsorbates.^34^ Conversely, a decrease in the *E*_ad_ of TMP from –1.2 eV to –0.82 eV, observed upon hydrolysis of the clean (001) facet (*i.e.* Ti_5C_ (001)–OH, Fig. 3a(iii)), can be supported by the NaOH treatment associated with the experimental upshift of δ^31^P from –29 ppm to –35 ppm (Ti_5C_ (001)–OH, Fig. 2a). Accordingly, the electronic effect of adsorbates on the *E*_ad_ of TMP on Ti_5C_ (001) matches the order derived experimentally: –F > –SO_4_ > –O– > –OH, within experimental error. Similar trends, but with a lower calculated *E*_ad_ of TMP for each adsorbate, can be found for Ti_5C_ (101) (Fig. 3b).

The DFT *E*_ad_ calculations of TMP on Ti_5C_ (001) with different levels of F coverage were carried out as an example to study the effect of surface coverage on the *E*_ad_ of TMP. As shown in Fig. S13,† the attachment of F on the (001) facet significantly enhances the calculated *E*_ad_ of TMP on the neighbouring Ti_5C_ (001), from –1.2 eV (Fig. S13(i)†) to –1.73 eV (Fig. S13(ii)†). However, the calculated *E*_ad_ only increases slightly (<0.05 eV) when doubling (Fig. S13(iii)†) or tripling (Fig. S13(iv)†) the F coverage in the surrounding area; this is not clearly distinguishable by ^31^P MAS NMR, as shown in Fig. S10.† In stark contrast, the calculated *E*_ad_ varies a lot with the electron affinity of the adsorbates on the (001) facet (Table S5†), –F (–1.76 eV), –SO_4_ (–1.51 eV), –O– (–1.2 eV) and –OH (–0.82 eV), and can be easily differentiated by ^31^P MAS NMR. This calculation result thus supports our experimental result that the electron affinity of the adsorbates (*i.e.* F > SO_4_ > OH) may override the role of the corresponding coverage in tuning the chemical state of Ti_5C_.

**Fig. 4:**
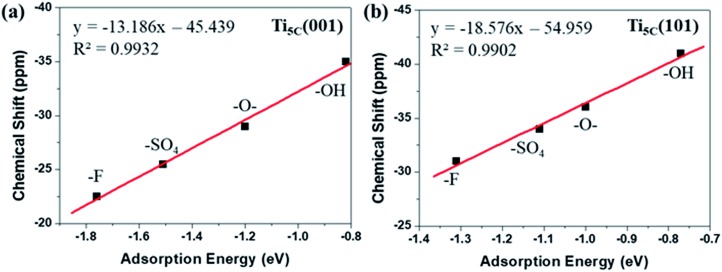
A linear regression plot using experimental δ^31^P (ppm) and calculated adsorption energy (eV) on the (a) (001) and (b) (101) facets of TiO_2_, promoted by –O–, –F, –OH and –SO_4_ groups (also see Tables S5 and S6†).

It has been observed that the *E*_ad_ of TMP molecules on Lewis acid sites shows a strong correlation with the NMR chemical shift value.^4^,^6^,^33^,^35^ Indeed, it is found that the calculated *E*_ad_ displays an excellent linear relationship with the experimental chemical shift value obtained for both the (001) (Fig. 4a) and (101) (Fig. 4b) facets promoted by various –O–, –F, –OH and –SO_4_ groups. Noticeably, different *y*-axis intercepts were obtained for Ti_5C_ (001) (–45.439 ppm) and Ti_5C_ (101) (–54.959 ppm) when the *E*_ad_ of TMP is zero on these two facets. According to DFT calculations from the literature, the (101) surface couples strongly with the bulk and can be regarded as an extension of the bulk state, while the (001) surface strongly deviates from the bulk due to the surface stress-induced orbital interactions, which thus gives rise to its unique reactivity.^36^ As the stronger TMP–Ti bond formation would push δ^31^P downfield, the ∼9.5 ppm difference can be attributed to the intrinsic electronic structure of the (001) and (101) facets. Here, the unusual strong molecular affinity of Ti_5C_ on the (001) facet (*cf.* Ti_5C_ on the (101) facet) is, for the first time, experimentally observed by this technique within the measurement errors; this may explain the calculated dissociative adsorption of water on the (001) facet (*cf.* associative adsorption on the (101) facet).^37^,^38^

### Pechmann condensation reaction

**Fig. 5:**
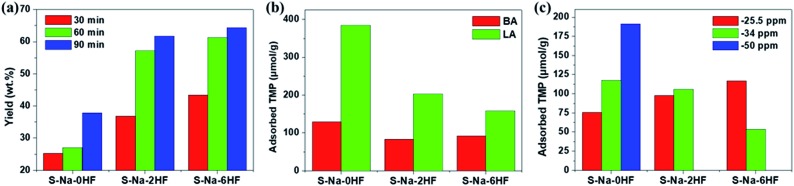
(a) Activity in the Pechmann condensation reaction over 30, 60 and 90 min, (b) the corresponding overall concentration of BA/LA sites and (c) the distribution of LA sites of various strengths for the catalysts S–Na–0HF/2HF/6HF. The quantitative results presented in (b) and (c) were calculated from the area of each deconvoluted peak in spectra in the third row of Fig. 2.

To demonstrate the importance of qualitative and quantitative assessment of the electronic states of facets in heterogeneous catalysis, the activities of S–Na–0HF/2HF/6HF for catalytic Pechmann condensation, using phloroglucinol and ethyl acetoacetate as starting reagents, were determined and the results are shown in Fig. 5a. In view of the Pechmann reaction, the condensation of phenol and a β-keto ester can be catalyzed by either BA or LA sites, and proceeds *via* transesterification, followed by intramolecular hydroalkylation and dehydration.^39^ As shown in Fig. 5a, the yield of 5,7-dihydroxy-4-methyl coumarin increases in the order S–Na–6HF > S–Na–2HF > S–Na–0HF over a 90 min reaction period. The total concentration of either BA or LA sites doesn’t seem to play the key role in this reaction, as S–Na–0HF with the highest total concentration of both BA and LA sites (Fig. 5b) shows the lowest yield among the three samples over the whole reaction time. As a similar BA strength is obtained for all three samples (–4 ppm, third row of Fig. 2), the strength of the BA does not seem to be the main factor for this catalyzed reaction. The concentration and distribution of LA sites with different strengths in S–Na–0HF/2HF/6HF are summarized in Fig. 5c. Since the stronger TMP–Ti bond formation pushes δ^31^P downfield, the corresponding acid strength of these three LA sites is in the order Ti_5C_ (001)–SO_4_ (–25.5 ppm) > Ti_5C_ (101)–SO_4_ (–34 ppm) > Ti_5C_ (101)–OH (–50 ppm). All three samples have both Ti_5C_ (001)–SO_4_ and Ti_5C_ (101)–SO_4_ sites, while S–Na–0HF has an extra weak LA site, Ti_5C_ (101)–OH. However, only the concentration of the Ti_5C_ (001)–SO_4_ site (–25.5 ppm) is found to be in the order S–Na–6HF > S–Na–2HF > S–Na–0HF (Fig. 5c), which is in accordance with the order of the product yield, which suggests that the specific concentration of this strong acid site is the main contributing factor for catalyzing this reaction in solution. Note that the strength of this LA site (–25.5 ppm) is comparable to that of sulfated/BF_3_-modified metal oxides (SO_4_^2–^/ZrO_2_ (ref. 40) and BF_3_/Al_2_O_3_ (ref. 41)), and is characteristic of super Lewis acidity.

## Conclusions

In summary, we have successfully applied an approach, which is a combination of chemical probe-assisted NMR and DFT modelling, to distinguish the chemical states of surface Ti on the (001) and (101) facets associated with –O–, –F, –OH and –SO_4_ surface groups on our synthesised TiO_2_ samples, within experimental error. This method seems to be tolerant to our synthesised nano-TiO_2_ surfaces, which are not as perfectly clean or perfectly smooth as those of large single crystals (which are free from any type of corrugation and additional deviations from the defined surface structures). It is clearly demonstrated that the electron affinity of these surface Ti states imposed by adsorbates (*i.e.* F > SO_4_ > OH) could vary between the facets and override the role of the corresponding coverage in tuning the chemical state of Ti_5C_. Also, the powerful traditional technique, XPS, was shown to only provide very limited information about the chemical state of surface cations, and in particular no information about their distribution among the facets or the disturbance from surface adsorbates was available. This probe-assisted NMR thus shows its potential in distinguishing the chemical states of surface cations on facets promoted with various surface groups, and we believe it is also applicable to other metal oxides and should enable systematic investigation of facet-dependent physiochemical properties in the future.

## Conflicts of interest

There are no conflicts to declare.

## Supplementary Material

Supplementary information
