# Supplementary material for: Differentiating surface titanium chemical states of anatase TiO2 functionalized with various groups
Source: Chem Sci. 2018 Jan 29;9(9):2493–500. doi: 10.1039/c7sc04828a (PMC5909675; doi:10.1039/c7sc04828a)
Supplement: Supplementary file 1 [file SC-009-C7SC04828A-s001.pdf]

Electronic Supplementary Material (ESI) for Chemical Science.
This journal is © The Royal Society of Chemistry 2018

Supporting Information for

Differentiating surface titanium chemical states of anatase TiO_2 functionalized with various surface groups

Yung-Kang Peng,[a] Hung-Lung Chou*,[b] and Shik Chi Edman Tsang*,[a]

[a]The Wolfson Catalysis Centre, Department of Chemistry, University of Oxford, Oxford, OX1 3QR, UK

[b]Graduate Institute of Applied Science and Technology, National Taiwan University of Science and Technology, Taipei 10617, Taiwan

*Correspondence: edman.tsang@chem.ox.ac.uk; HLCHOU@mail.ntust.edu.tw

Experimental details

Synthesis of 6HF, 2HF and 0HF TiO_2 samples. 5.0 mL of $Ti(OC_4H_9)_4$ was mixed with a certain amount of hydrofluoric acid (40~48 wt.%) in a Teflon-lined autoclave with a capacity of 40 mL and subsequently heated to 180 °C at a ramp rate of 2°C·min^{-1}. The temperature was kept at 180 °C for 24 h.[1] After hydrothermal reaction, the white precipitate was collected, washed with ethanol and distilled water three times, and then dried in an oven at 80 °C overnight. 0.6 mL and 0.2 mL of hydrofluoric acid was added for 6HF and 2HF samples, respectively, while, instead of hydrofluoric acid, 0.6 mL of H_2O was employed for 0HF sample.

NaOH removal of surface fluorine. The NaOH wash was employed in this study according to previous literature.[1] 1.0 g of as-prepared TiO_2 sample was treated in 50.0 mL of 0.1 M NaOH solution under magnetic stirring for 10 h. The solid sample was then washed with distilled water several times until neutral. After centrifugation, the solid was dried at 80 °C overnight. TiO_2 samples (i.e. 0HF, 2HF and 6HF) obtained after washing 0.1M NaOH wash were denoted as Na-0HF, Na-2HF and Na-6HF.

Sulfation of TiO_2. To obtain the sulfated TiO_2, ammonium sulphate ($(NH_4)_2SO_4$) was utilized as the precursor of sulfate during catalyst preparation.[2] For example, 1.0 g of NaOH wahsed sample was added into 10.0 mL of 1 mol.L^{-1} $(NH_4)_2SO_4$ solution to stir for 8 h and then collected by centrifugation. The solid was then calcined at 450 °C for 4 h. We denoted these samples as S-Na-0HF, S-Na-2HF and S-Na-6HF.

Pechmann condensation testing. The activities of TiO_2 samples were tested for the synthesis of 5,7-dihydroxy-4-methyl coumarin from phloroglucinol and ethyl acetoacetate under solvent-free conditions. 5 mmol of phloroglucinol was reacted with 10 mmol of ethyl acetoacetate in the presence of 100 mg of catalyst. The reaction mixture was kept at 130 °C under reflux for a 30, 60 and 90 minutes. The reaction mixture was cooled down and filtered to separate the catalyst. The product was analysed by liquid chromatography (DIONEX U3000). The yield of 5,7-dihydroxy-4-methyl coumarin was calculated as: Yield (%) = (Obtained weight of product)/(Theoretical weight of product)×100.

Raman measurement. Raman spectra were measured with via Raman Microscope (Renishaw) with a laser excitation wavelength of 532 nm. Exposure time of 10 sec and 8 number scans were adopted for each measurement.

XPS measurement. XPS measurements were recorded on a Thermo Scientific K-Alfa XPS instrument equipped with micro-focused monochromated Al X-ray source. The source was operated at 12 keV and a 400 micron spot size was used. The analyzer operated at the analyzer energy (CAE) of 200 eV for survey scans and 50 eV for detailed scans. Charge neutralization was applied using a combined low energy/ ion flood source. The data acquisition and analysis were conducted with CasaXPS (Casa software Ltd.). The peak position was referenced to C1s peak of the carbon tape at 285.00 eV.

TMP-adsorbed sample preparation. About 150 mg of TiO_2 sample was placed in a home-made glass tube and activated at 150 °C for 2 h under vacuum (10^{-1} Pa). After cooling down to room temperature, 300 μmol/catalyst g (calculated by the pressure and volume of isolated system) of TMP was then introduced. Wait ~10 min for the reach of equilibrium between TMP and catalyst surface. Extra TMP molecules were removed by vacuum system. These steps were repeated three times to ensure the fully adsorption of TMP on catalyst surface. The sample tube was then flame sealed for storage and transferred to Bruker 4 mm ZrO_2 rotor with a Kel-F endcap in a glove box under nitrogen atmosphere before NMR measurement. See our previous report for more details.[3.4]

NMR measurement. The solid state magic angle spinning (MAS) NMR experiments were carried out using a Bruker Avance III 400WB spectrometer at room temperature. Magic angle spinning speed of 12 kHz and high power decoupling (HPDEC) sequence were adopted here. Considering the long relaxation time of ^{31}P nuclei in NMR experiment, 30° pulse with the width of 1.20 μs, 15 s delay time was used. The radiofrequency for decoupling was 59 kHz. The spectral width was 400 ppm, from 200 to −200 ppm. The number of scanning was 800. The ^{31}P chemical shifts were reported relative to 85% aqueous solution of H_3PO_4, with $NH_4H_2PO_4$ as a secondary standard (0.81 ppm). See our previous report for more details.[3.4]

NMR spectrum deconvolution. All raw TMP NMR spectra were deconvoluted using the software 'peakfit v4.12'. We employed "gauss area" and ensured all results with R^2 value > 0.98. Notice that the raw spectra data of samples with the same treatment/modification show a standard deviation of ±1 ppm in chemical shift position. For example, during the spectral deconvolution of 2HF and 6HF samples, we have fixed two positions of Ti_{5c}(101) and Ti_{5c}(001) at -31 ppm and -22.5 ppm within ±1 ppm uncertainty. Also, for S-Na-PD, S-Na-(101) and S-Na-(001), the position of Ti_{5c}(101) and Ti_{5c}(001) were fixed at -25.5 and -34 ppm within ±1 ppm uncertainty. See ref. 4 for details spectrum deconvolution.

DFT calculations. Projector-augmented waves (PAW)[5,6] generalized gradient approximation (GGA)[7] was employed in DFT calculations. In the plane wave calculations, cutoff energy of 500 eV was applied and automatically set by the total energy convergence calculation for anatase $TiO_2(001)$ and $TiO_2(101)$ slab system. The primitive unit cell of TiO_2 was constructed to consist of tetragonal anatase TiO_2 structure containing eight O atoms with four Ti atoms; the system was then allowed to reach its lowest energy configuration by a relaxation procedure. The k-point grid determined by the Monkhorst-Pack method was 7 × 7 × 3 for bulk calculations in this study. The calculated lattice parameters of TiO_2 were 3.776 × 3.776 × 9.486 Å, which was in good agreement with the experimental value (3.785 × 3.785 × 9.514 Å).[8] For the modeling of both (001) and (101) surfaces, we adopted a slab containing twelve layers of Ti-O units. The surface was constructed as a slab within the three dimensional periodic boundary conditions. This model was separated from their images in the direction perpendicular to the surface by a 14 Å vacuum space. The bottom three layers were kept fixed to the bulk coordinates; full atomic relaxations were allowed for the top nine layers. During calculations, a 3 × 3 × 1 *k*-Point mesh was used in the 4 × 4 super cell. A suitable dimension of supercell (11.328 × 11.328 × 26.255 $Å^3$) was found to perform the adsorption of trimethylphosphine (TMP) on $TiO_2(001)$. Supercell with dimension 10.885 × 11.328 × 23.353 $Å^3$ was used for $TiO_2(101)$. The atoms in the cell were allowed to relax until the forces on unconstrained atoms were less than 0.01 eV/Å. The adsorption energy, E_{ad}, is defined as the sum of interactions between the capping molecule and slab system, and it is given as $E_{ad} = E_{total} - E_{TiO2(001)} - E_{TMP}$, where E_{total}, $E_{TiO2(001)}$ and E_{TMP} are the energy of total system, $TiO_2(001)$ slab and TMP molecule, respectively. The negative sign of E_{ads} corresponds to the energy gain of the system due to molecular adsorption. The calculation of TMP-$TiO_2(101)$ was carried out similarly. To calculate the effects of various adsorbates to surface Ti chemical states among facets, we placed F-, OH-, SO_4 molecules on $TiO_2(001)$ and $TiO_2(101)$ and then calculated the corresponding E_{ad} of the TMP on the given surfaces. For the F case, two H (for charge balance) and two F atoms were introduced on both the $TiO_2(001)$ and $TiO_2(101)$ slabs. 1~3 F atoms were also placed on $TiO_2(001)$ slab to evaluate the coverage effect on the E_{ad} of TMP. For the OH case, two OH groups were generated by the surface hydrolysis of Ti-O-Ti bonds on both slabs. For the SO_4 case, two H atoms (for charge balance) and a SO_4 molecule binds to two Ti_{5C} sites via its two oxygen atoms were introduced on both the slabs. To simulate solid-state NMR environment, the plane wave DFT code, VASP, was also adopted to calculate adsorption energies for TiO_2 systems using the linear response method.[9,10] The five

INCAR tags relevant to linear response calculation of TiO_2 (001) and (101) systems are shown below:

ICHIMAG=.TRUE.

QD=0.001

ICHIBARE=1

LNMR_SYM_RED=.FALSE.

NLSPLINE=.TRUE.

All simulation graphics in this work were generated using GaussView version 3.0.

Degree of surface F/SO_4 modification

The calculation of F-modified TiO_2 (6HF) case are elaborated as an example below:

According to Scheme 1b, the concentration of F attached Ti_{5C} can be obtained 1 : 1 from the protonation of TMP by neighboring Brönsted acid proton. Therefore, the total concentration of F-Ti_{5C} on (001) and (101) facets of 6HF sample is 89.4 μmol/g (Table S3).

The concentration of rest surface Ti_{5C} atoms (without F attachment) can be obtained from the NMR signal in Lewis acid range (the formation of TMP-Ti_{5C}, Table S3) including Ti_{5C}(001), Ti_{5C}(101) and surface oxygen vacancy: 53.2 + 32.0 + 33.1 = 118.3 μmol/g.

Total concentration of surface Ti_{5C} on 6HF sample: 89.4 + 118.3 = 207.7 μmol/g.

As F won't bind to surface Ti_{6C}, O_{2C} and O_{3C}, the percentage of F attached Ti_{5C} on 6HF sample is thus 89.4/207.7 = 43.0%.

By adopting similar calculation to the other samples, the total concentrations of surface Ti_{5C} and the surface F/SO_4 coverage of 6HF and 2HF samples can be obtained and are summarized in Table S4. Noted that S-Na-0HF wasn't consider here due to the generation of Ti_{5C}(101)-OH during the extensive hydrolysis.

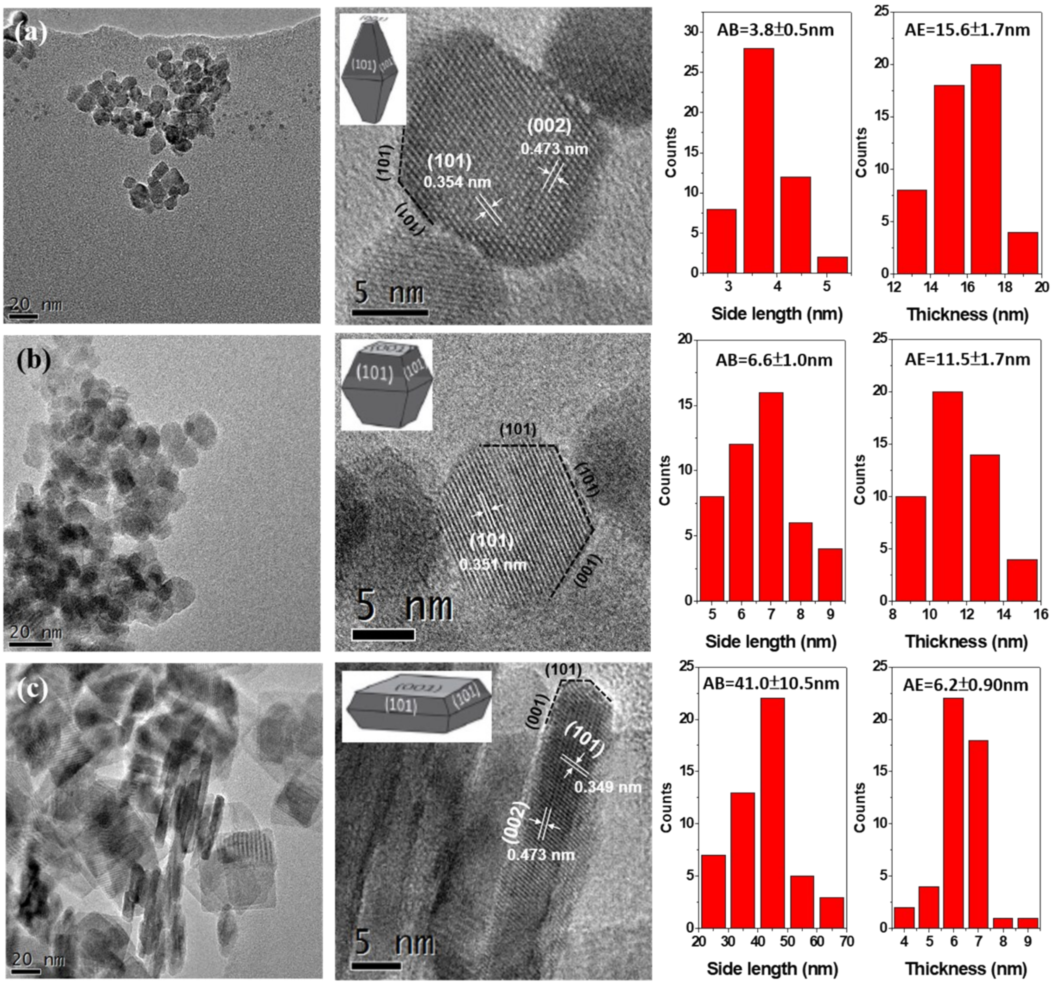

Figure S1. TEM (first column) and HRTEM (second column) images of as-prepared (a) 0HF, (b) 2HF, and (c) 6HF and their corresponding AB (face length) and AE (thickness) value (see Figure S3 for the surface area calculation of (001) and (101) facets) (50 particles are used in each histogram).[4]

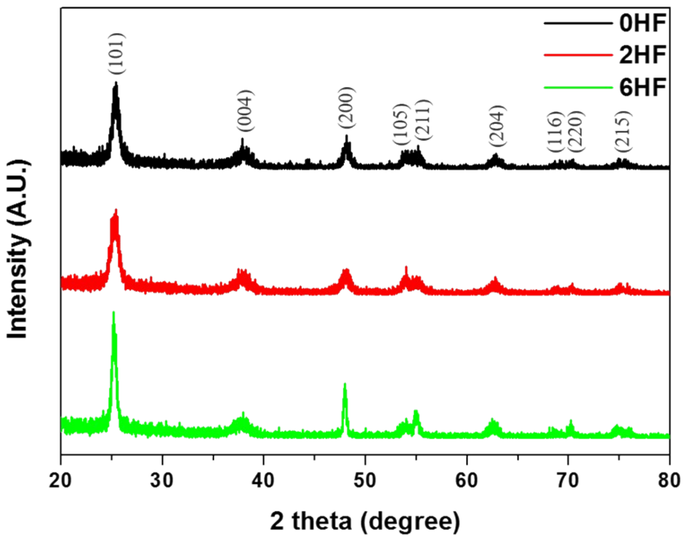

Figure S2. XRD spectra of as-prepared anatase 0HF, 2HF and 6HF.

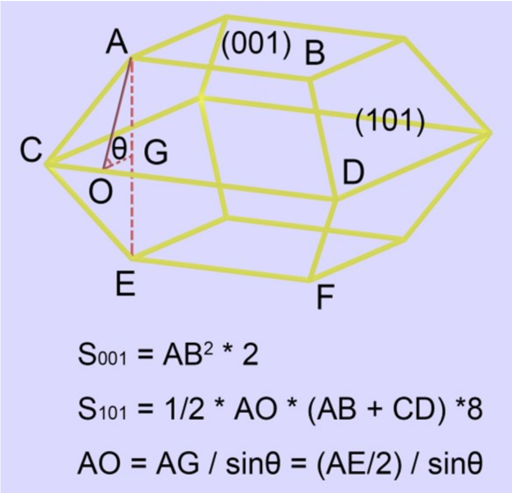

Figure S3. Simulated shape of the TiO_2 anatase single crystal and the equation for the surface area calculation of (001) and (101) facets (AB and CD are considered of the same value as face length herein; AE is equal to the thickness, θ of 68.3° is the angle between (001) and (101)).[2]

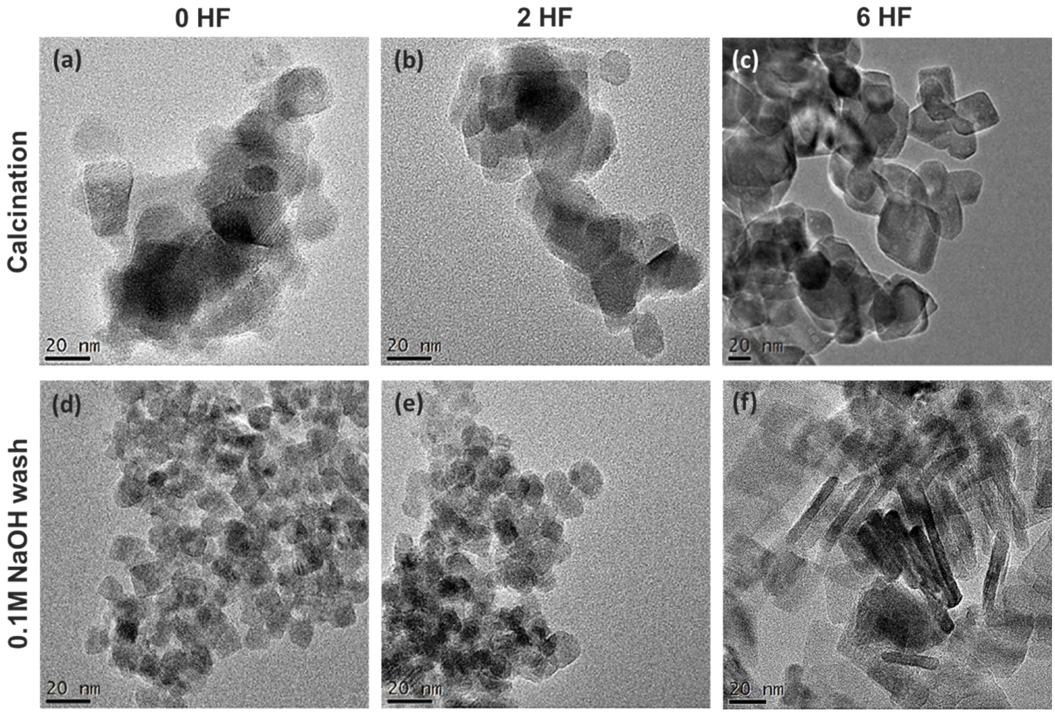

Figure S4. TEM images of TiO_2 samples after (a–c) calcination treatment: (a) Cal-0HF, (b) Cal-2HF, (c) Cal-6HF and (d–f) NaOH wash: (d) Na-0HF, (e) Na-2HF, (f) Na-6HF.

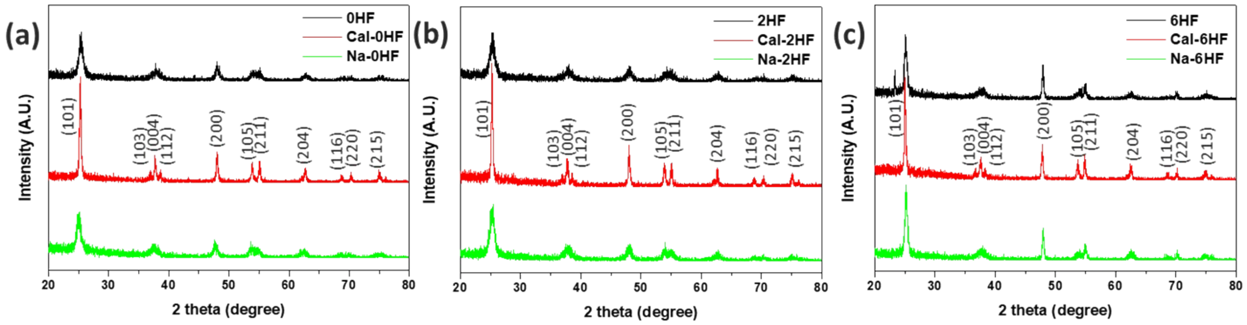

Figure S5. XRD spectra of as-prepared (a) 0HF, (b) 2HF and (c) 6HF TiO_2 samples with different post-treatments (calcination and NaOH wash).[4]

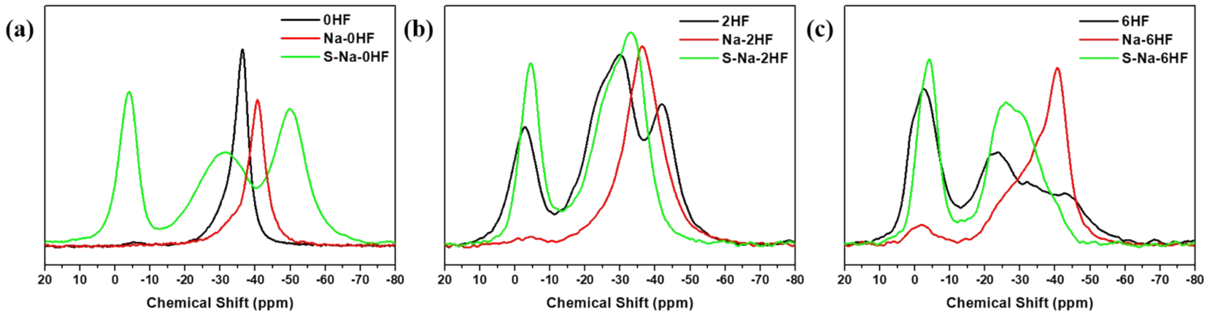

Figure S6. ^{31}P MAS NMR spectra of TMP-adsorbed (a) 0HF, (b) 2HF and (c) 6HF samples (black line) with NaOH wash (red line, i.e. Na-0HF/2HF/6HF) followed by sulfate modification (green line i.e. S-Na-0HF/2HF/6HF).

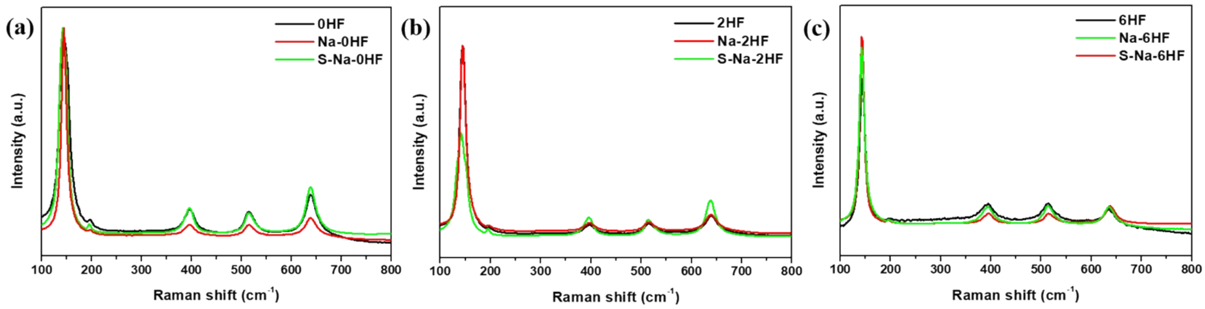

Figure S7. Raman spectra of (a) 0HF, (b) 2HF and (c) 6HF samples (black line) with NaOH wash (red line, i.e. Na-0HF/2HF/6HF) followed by sulfate modification (green line i.e. S-Na-0HF/2HF/6HF).

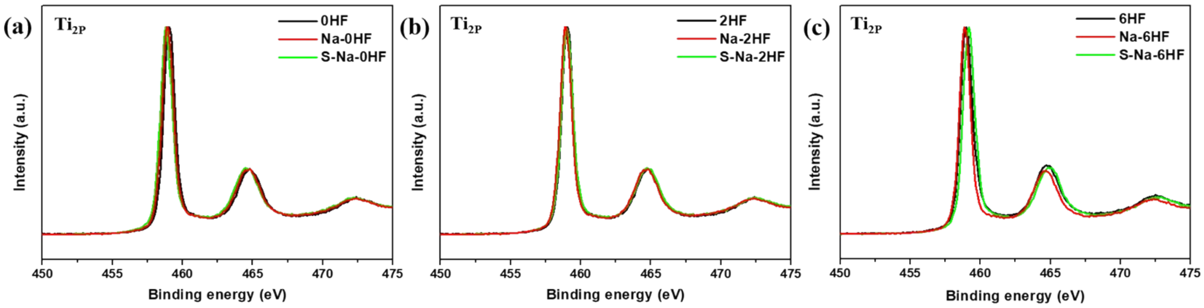

Figure S8. XPS Ti_{2P} spectra of (a) 0HF, (b) 2HF and (c) 6HF samples (black line) with NaOH wash (red line, i.e. Na-0HF/2HF/6HF) followed by sulfate modification (green line i.e. S-Na-0HF/2HF/6HF).

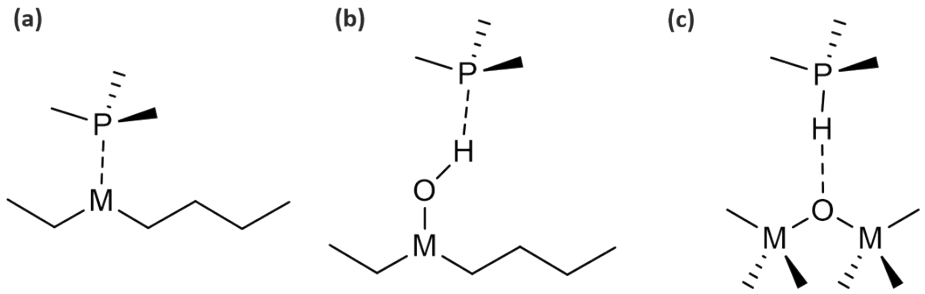

Figure S9. TMP molecule interacts (a) with metal cation LA center; (b) with hydroxyl proton LA center (hydrogen bonding interaction); (c) on bridging hydroxyl proton (Brønsted acid, BA) site, the formation of $TMPH^+$ complex).

Figure S9 shows three scenarios of interactions between TMP and metal oxide: (a) with metal cation LA center; (b) with hydroxyl proton LA center (hydrogen bonding interaction); (c) on bridging hydroxyl proton (Brønsted acid, BA) site, the formation of $TMPH^+$ complex). The $\delta^{31}P$ of adsorbed TMP spans over a wide range (-20~-58 ppm) when interacting with various metal cations on different solid acids (Figure S9a), whereas a $TMPH^+$ ionic complex formed when a TMP molecule adsorbs onto a bridging hydroxyl proton tends to give rise to a ^{31}P resonance in a much narrower range of -2 to -5 ppm (Figure S9c).[11] Therefore, Brønsted (proton donor) and Lewis acid (electron acceptor) sites presented in a solid acid catalyst can be readily distinguished using ^{31}P NMR of adsorbed TMP. On the other hand, TMP on an isolated hydroxyl proton surface usually gives a signal at higher field (~-61 ppm, Figure S9b).[11]

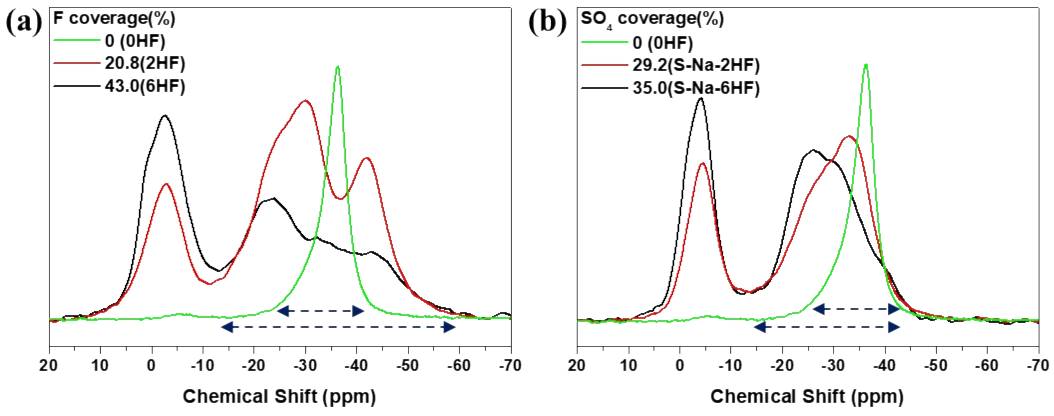

Figure S10. Comparison of TMP-adsorbed 0HF, 2HF and 6HF samples with various surface coverage of (a) F and (b) SO_4. Their coverages are summarized inTable S4.

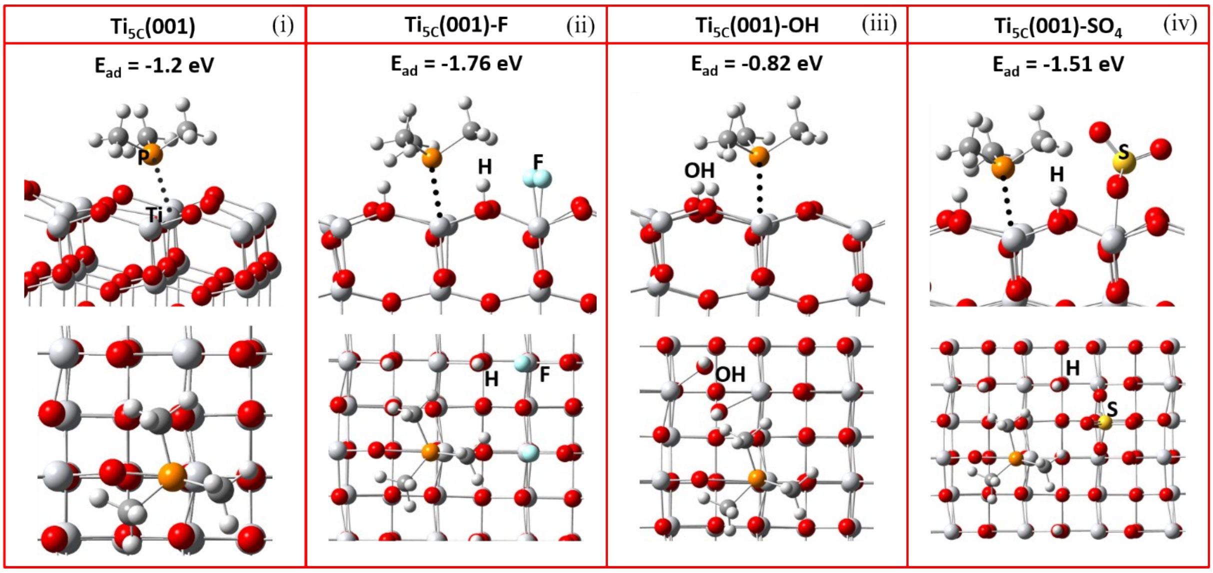

Figure S11. Schematic side (first row) and top (second row) view of molecular interaction and corresponding calculated adsorption energy (E_{ad}) between TMP and Ti_{5C}(001) promoted with (i) -O-, (ii) -F, (iii) -OH and (iv) -SO_4 groups. (Ti: light grey; O: red; P: orange; C: grey; H: white; S: yellow).

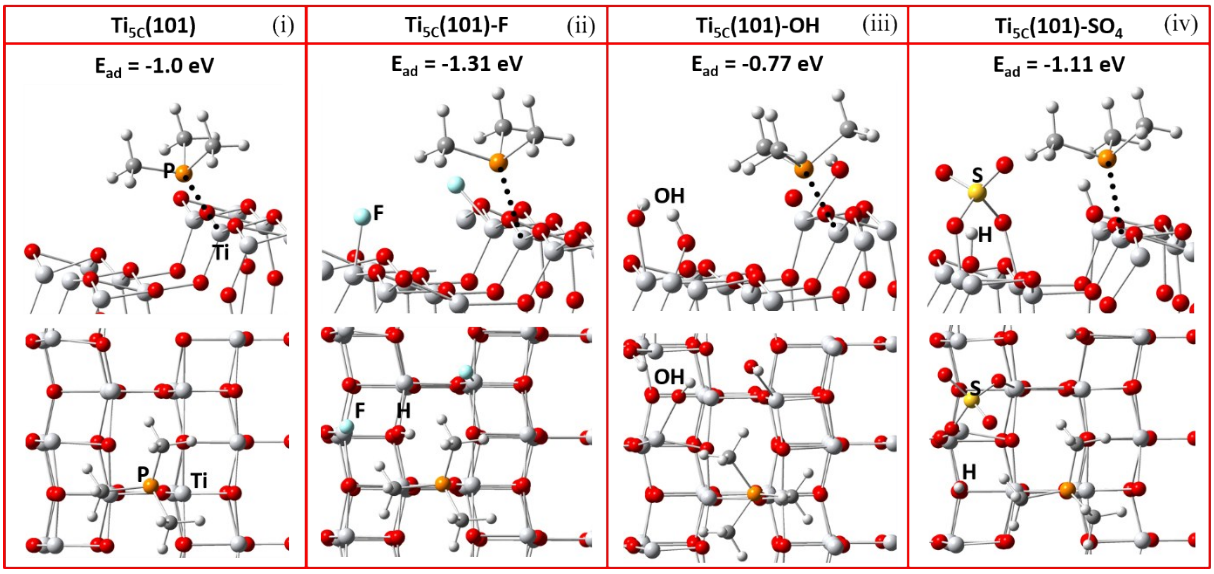

Figure S12. Schematic side (first row) and top (second row) view of molecular interaction and corresponding calculated adsorption energy (E_{ad}) between TMP and Ti_{5C}(101) promoted with (i) -O-, (ii) -F, (iii) -OH and (iv) -SO_4 groups. (Ti: light grey; O: red; P: orange; C: grey; H: white; S: yellow).

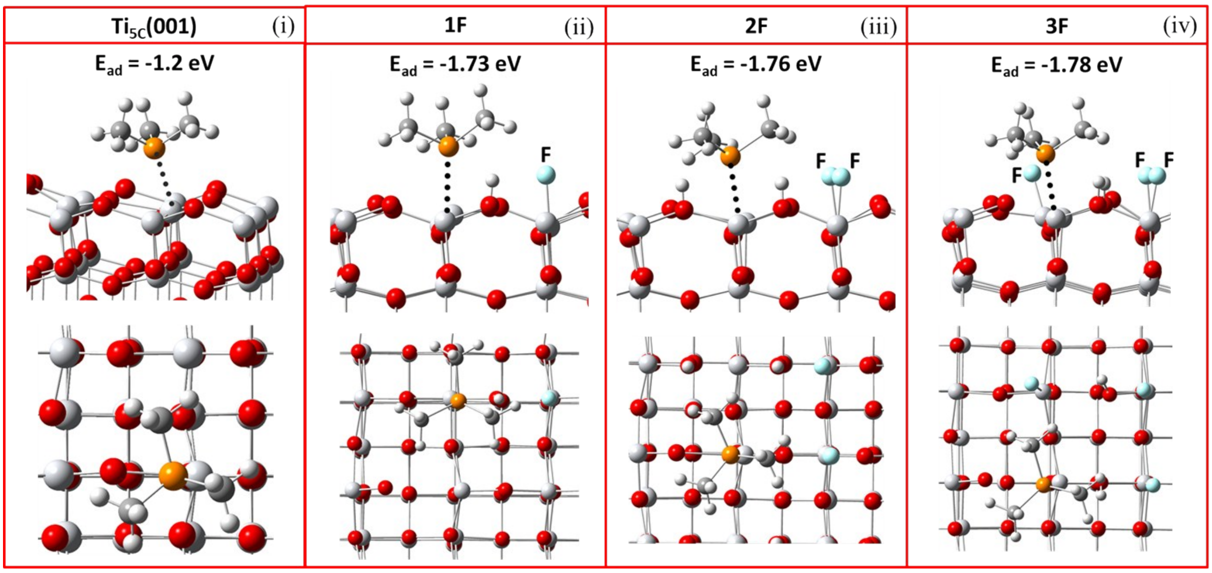

Figure S13. Schematic side (first row) and top (second row) view of molecular interaction and corresponding calculated adsorption energy (E_{ad}) between TMP and Ti_{5C}(001) (i) without F and promoted with (ii) 1F, (iii) 2F, (iv) 3F atoms. (Ti: light grey; O: red; P: orange; C: grey; H: white).

Table S1. Samples preparation conditions and the calculated percentage of exposed (101) and (001) facet.

Sample	Solvent	Face length (AB, nm)	Thickness (AE, nm)	% (101)	% (001)
0HF	6 mL H_2O	3.8 ± 0.5	15.6 ± 1.7	89.8	10.2
2HF	2 mL HF/4 mL H_2O	6.6 ± 1.0	11.5 ± 1.7	78.9	21.1
6HF	6 mL HF	41.0 ± 10.5	6.2 ± 0.9	24.6	75.4

Table S2. Particle size (calculated from the full width at half-maximum of the (101) peak in Figure S5 using Scherrer equation) and BET surface area data of 0HF, 2HF, 6HF and their corresponding calcination(Cal-)/NaOH wash(Na-) treatments.

Sample	Particle size (nm)	BET (m^2/g)
0HF	10.3	123.3
Cal-0HF	25.8	15.8
Na-0HF	9.9	145.4
2HF	8.2	163.0
Cal-2HF	28.4	40.8
Na-2HF	8.7	152.4
6HF	15.6	83.0
Cal-6HF	29.7	29.9
Na-6HF	16.6	85.1

Table S3. Summary of the qualitative (chemical shift) and quantitative (peak area) of each deconvoluted peak in the region of Brønsted acid site (−2 to −5 ppm) and Lewis acid site (−20 to −58 ppm).[4] The concentration of adsorbed TMP on each site was calculated according to corresponding peak area (*Adsorbed TMP molecules in μmol/g)

Samples	BA (-2~-5 ppm)		LA sites (-20~-58 ppm)						
	ppm	Total TMP*	Ti_{5c}(001) (ppm)	TMP*	Ti_{5c}(101) (ppm)	TMP*	Others (ppm)	TMP*	Total TMP*
0HF	-	-	-29	106.8	-36	605.3	-	-	712.1
Na-0HF	-	-	-35	143.3	-41	393.9	-46	59.7	596.9
S-Na-0HF	-3.7	128.7	-25.5	75.6	-34	117.1	-50	191.2	383.9
2HF	-2.7	50.1	-22.5	72.4	-31	57.1	-42.5	60.9	190.4
Na-2HF	-2.9	5.8	-28	96.0	-36.5	222.6	-41	117.8	436.4
S-Na-2HF	-4.3	83.6	-25.5	97.5	-34	105.6	-	-	203.1
6HF	-2.6	89.4	-22.5	53.2	-31	32.0	-42.5	33.1	118.3
Na-6HF	-2.4	30.9	-28	135.1	-36.5	149.1	-41	181.6	465.8
S-Na-6HF	-3.7	91.4	-25.5	116.4	-34	53.6	-	-	170.0

Table S4. Summary of the total concentration of surface Ti_{5C} and the surface F/SO_4 coverage of 2HF and 6HF samples. [1]Concentration of F/SO_4 obtained by NMR BA signal (μmol/g). [2]Total concentration of surface Ti_{5C} obtained by NMR BA and LA signals (μmol/g). [3]Percentage of F/SO_4 coverage on surface Ti_{5C}.

Samples	Adsorbate	BA[1]	BA+LA[2]	Coverage[3]
6HF	F	89.4	207.7	43.0%
2HF	F	50.1	240.5	20.8%
S-Na-6HF	SO_4	91.4	261.4	35.0%
S-Na-2HF	SO_4	83.6	286.7	29.2%

Table S5. Experimental $\delta^{31}P$ and calculated adsorption energy of TMP (both PAW-GGA and linear reponse) on $Ti_{5C}(001)$ promoted with -F, $-SO_4$, -O- and -OH groups.

Structure	$E_{adsorption}$ (eV) PAW-GGA	$E_{adsorption}$ (eV) Linear response	$\delta^{31}P$ (ppm)
$Ti_{5C}(001)$-F	-1.76	-1.76	-22.5
$Ti_{5C}(001)$-SO_4	-1.51	-1.51	-25.5
$Ti_{5C}(001)$	-1.2	-1.2	-29
$Ti_{5C}(001)$-OH	-0.82	-0.82	-35

Table S6. Experimental $\delta^{31}P$ and calculated adsorption energy of TMP (both PAW-GGA and linear reponse) on $Ti_{5C}(101)$ promoted with -F, $-SO_4$, -O- and -OH groups.

Structure	$E_{adsorption}$ (eV) PAW-GGA	$E_{adsorption}$ (eV) Linear response	$\delta^{31}P$ (ppm)
$Ti_{5C}(101)$-F	-1.31	-1.31	-31
$Ti_{5C}(101)$-SO_4	-1.11	-1.11	-34
$Ti_{5C}(101)$	-1.0	-1.0	-36
$Ti_{5C}(101)$-OH	-0.77	-0.77	-41

References:

[1] H. G. Yang, G. Liu, S. Z. Qiao, C. H. Sun, Y. G. Jin, S. C. Smith, J. Zou, H. M. Cheng and G. Q. Lu, *J. Am. Chem. Soc.* 2009, **131**, 4078–4083.

[2] Y. Hu, B. Guo, Y. Fu, Y. Ren, G. Tang, X. Chen, B. Yue and H. He, *Chem. Commun.* 2015, **51**, 14219–14222.

[3] Y. -K. Peng, L. Ye, J. Qu, L. Zhang, Y. Fu, I. F. Teixeira, I. J. McPherson, H. He and S. C. E. Tsang, *J. Am. Chem. Soc.* 2016, **138**, 2225–2234.

[4] Y.-K. Peng, Y. Hu, H.-L. Chou, Y. Fu, I. F. Teixeira, L. Zhang, H. He and S. C. E. Tsang, *Nat. Commun.* 2017, **8**, 675.

[5] D. Vanderbilt, *Phys. Rev. B: Condens. Matter* 1990, **41**, 7892–7895.

[6] M. C. Payne, M. P. Teter, D. C. Allan, T. A. Arias and J. D. Joannopoulos, *Rev. Mod. Phys.* 1992, **64**, 1045–1097.

[7] J. P. Perdew, J. A. Chevary, S. H. Vosko, K. A. Jackson, M. R. Pederson, D. J. Singh and C. Fiolhais, *Phys. Rev. B: Condens. Matter* 1992, **46**, 6671–6687.

[8] D. A. H. Hanaor and C. C. Sorrell, *J. Mater. Sci.* 2011, **46**, 855–874.

[9] C. J. Pickard and F. Mauri, *Phys. Rev. B* 2001, **63**, 245101.

[10] J. R. Yates, C. J. Pickard and F. Mauri, *Phys. Rev. B* 2007, **76**, 024401.

[11] A. Zheng, S.-B. Liu and F. Deng, *Chem. Rev.* 2017, **117**, 12475−12531.
